# Neglect Patients Exhibit Egocentric or Allocentric Neglect for the Same Stimulus Contingent upon Task Demands

**DOI:** 10.1038/s41598-017-02047-x

**Published:** 2017-05-16

**Authors:** Louise-Ann Leyland, Hayward J. Godwin, Valerie Benson, Simon P. Liversedge

**Affiliations:** 1 School of Psychology and Clinical Language Sciences, University of Reading, Whiteknights Campus, Reading, RG6 7BE UK; 2 School of Psychology, University of Southampton, Highfield Campus, Southampton, SO17 1BJ UK

## Abstract

Hemispatial Neglect (HN) is a failure to allocate attention to a region of space opposite to where damage has occurred in the brain, usually the left side of space. It is widely documented that there are two types of neglect: *egocentric* neglect (neglect of information falling on the individual’s left side) and *allocentric* neglect (neglect of the left side of each object, regardless of the position of that object in relation to the individual). We set out to address whether neglect presentation could be modified from egocentric to allocentric through manipulating the task demands whilst keeping the physical stimulus constant by measuring the eye movement behaviour of a single group of neglect patients engaged in two different tasks (copying and tracing). Eye movements and behavioural data demonstrated that patients exhibited symptoms consistent with *egocentric* neglect in one task (tracing), and *allocentric* neglect in another task (copying), suggesting that task requirements may influence the nature of the neglect symptoms produced by the same individual. Different task demands may be able to explain differential neglect symptoms in some individuals.

## Introduction

## Stroke, Hemispatial Neglect and ‘Types’ of Neglect

Every year, approximately 15 million people worldwide suffer a stroke^1^. Most stroke survivors experience severe cognitive and physical disabilities resulting from brain damage that can persist for the remainder of their lives^2^ and stroke is the most common cause of chronic physical disability in adults^3^. Hemispatial neglect (HN) is a neuropsychological condition that frequently results from stroke^4^ in which the patient exhibits decreased awareness, i.e. neglect, of an area of space (usually the left side) that is opposite to the lesioned hemisphere (contralesional). Patients with HN will often leave food on the contralesional side of their plate, fail to apply make-up to/shave that side of their face, and do not respond to people situated within the neglected region^5^: put simply, it is as if that side of space has vanished from their internal representation of the world. Hemispatial neglect is a debilitating disorder which is a strong predictor of hampered functional recovery, and is also a major disruptive factor impeding rehabilitative success following stroke^6–8^. Despite its prevalence, neglect remains a very poorly understood phenomenon. Developing understanding of the nature of HN, and the factors that affect it, is critical for patient diagnosis and development of remediation treatments. It also provides direct insight into how the healthy brain processes spatial information^9^.

It is widely documented that there are two types of neglect: *egocentric* and *allocentric*. Egocentric neglect is apparent when objects are neglected relative to the encoder’s own position (e.g., objects that fall to the left side of the encoder), whereas allocentric neglect involves neglect occurring in relation to the objet itself, regardless of its position relative to the encoder (e.g., the left side of an object would be neglected even if the object itself was entirely located on the right side of the encoder).

Clinicians and research scientists have argued that these different types of neglect result from disruption to different regions of the brain. Damage to the dorsal stream of visual processing, specifically the right supramarginal gyrus, has been associated with egocentric neglect, and disruption to the ventral streams of visual processing, particularly the posterior inferior temporal gyrus, with allocentric neglect^10^. To this extent, these categories of HN are frequently considered to be mutually exclusive. Accordingly, many studies have used a between participant design to investigate these forms of neglect^11–15^ with few studies using a within participant design to assess factors, for example task requirements, that may affect the presentation of the type of neglect (i.e., whether it is egocentric or allocentric neglect)^16^. A consequence of this approach is that often studies have not directly investigated the impact of task demands on the type of neglect presentation within the same patient when presented with the same stimulus. Here we directly address this issue, demonstrating that the specific demands of two similar tasks, namely, copying a figure and tracing a figure, cause the same participants to exhibit behaviour consistent with egocentric neglect when tracing and allocentric neglect when copying.

In a relevant study, Karnath and Niemeier^16^ employed a visual search task to investigate whether varying task instructions for three patients with left neglect affected the type of neglect they exhibited. Eye movements were recorded to examine inspection patterns during search for a target letter across a stimulus set comprised of multiple different letters. Importantly, the visual array was either categorised as homogeneous (where all the letters comprising the search array were the same colour) or segmented (where the letters comprising the search array were presented in vertical bands of different colours, either with or without a visible line demarcating the boundaries of the bands). Patients were required to undertake target search in the homogeneous array and subsequently to restrict their search in the segmented condition to one of the bands denoted by letters being a particular colour. Karnath and Niemeier found that when each participant searched the homogeneous visual array, the left half of the entire array was neglected. However, in the segmented condition, participants only failed to inspect the left side of the band of coloured letters to which they had been directed. Therefore, in the segmented condition patients neglected an area of space that was previously attended to and searched effectively in the homogenous condition.

Although Karnath and Niemeier’s findings are interesting, there are several confounds within their experimental design that bring their conclusions into question. First, the working memory and visual processing loads are clearly not consistent for the two types of stimuli in the search task, as, for example, a smaller region of space is required to be searched in the segmented condition. Second, it is unclear whether the change in the nature of the task, or instead, the change in the nature of the stimulus (colouring of the letters and presenting clear boundaries for the regions) brought about the effects that they observed. It is very well documented that the basic visual properties of a stimulus fundamentally influence the manner in which that stimulus is visually processed (e.g., how we process illusory objects such as Kanisza squares^17^; the efficiency and automaticity with which we process colour^18, 19^). Furthermore, it is also the case that specifically instructing patients as to how they should allocate their visual attention during search fundamentally alters their search behaviour^17^. A critical point to note here is that it is impossible to evaluate the strength of Karnath and Niemeier’s claims when experimental confounds exist. Evidence that would be far more compelling in relation to this issue would derive from experimental stimuli that were identical under different experimental conditions, and, for which task instructions did not explicitly direct patients in how they should allocate their attention.

For these reasons, we undertook an investigation of whether the presentation of neglect (i.e., egocentric or allocentric) could be manipulated by implicit task demands within patients who all consistently exhibited classic egocentric patterns of neglect as indexed by standardised tasks. Furthermore, in our eye movement experiment we used stimuli that were identical in terms of their visual information, but asked participants to view those stimuli under different experimental conditions. Doing so enabled us to circumvent confounds surrounding symptomatic behavioural changes in our patients that may have arisen due to differences in the physical properties of the stimuli. Finally, and very importantly, we employed task instructions that did not explicitly direct patients to allocate their attention differently under different experimental conditions. Instead, our task instructions under the different experimental conditions were such that they implicitly required patients to differentially allocate their attention with respect to the stimulus in order to succeed in their task. In our study, we simply required patients to either trace or copy a picture and we tracked their eye movements as they undertook these tasks. Eye movement recording provides an excellent online method to investigate the moment-to-moment visual, cognitive and attentional processes that occur during completion of a task^20^. Also, eye tracking often provides a more sensitive measure of spatial deficits than do simple behavioural responses^16^.

**Figure 1:**
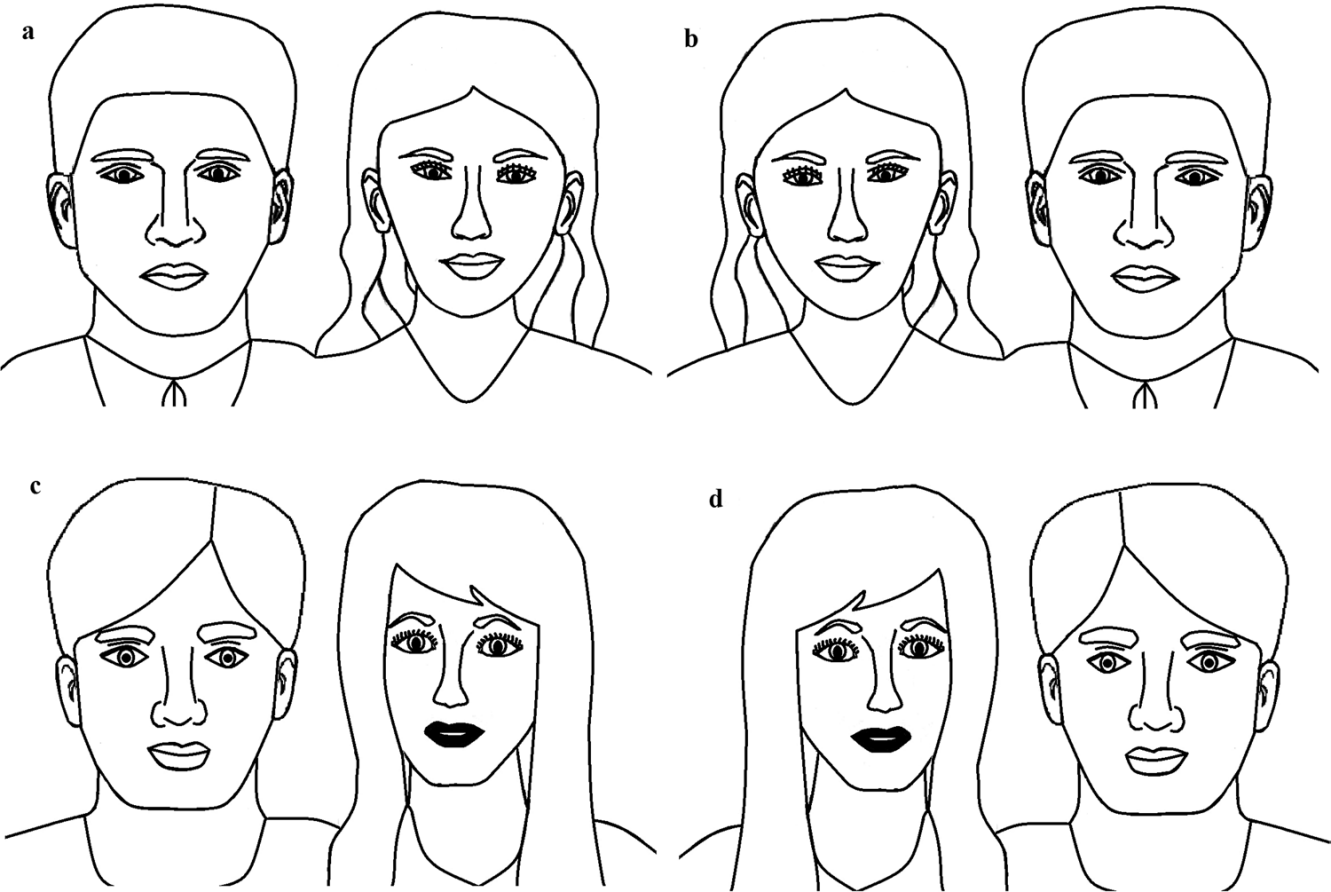
Figures used in the Tracing and Copying Conditions. The participants either traced or copied (**a**) and (**d**) or (**b**) and (**c**).

In our experiment we employed two identical sets of stimuli for two conditions: a Copying Condition and a Tracing Condition. The stimuli that were to be copied or traced took the form of outline drawings of two horizontally adjacent human heads and shoulders (one male, one female, see Fig. 1). During copying, successive processing episodes are required in which a portion of the visual array is encoded and then represented in memory, after which a representation corresponding to that stored in memory is reproduced on the page^21, 22^. Through multiple episodes, a copier incrementally develops a replica of the stimulus. Critically, in this situation the copier must encode and represent the spatial relations that exist in, and between, the component parts of the stimulus image. Ordinarily, attention will be allocated to each meaningful portion of the stimulus sequentially. For example, the copier might first produce a copy of the head and shoulders on the right, and then, after the rightward stimulus has been copied to satisfaction, they might then produce a copy of the head and shoulders on the left. To be clear, when copying such a stimulus, it is extremely unlikely that the copier would produce a small portion of the right head, then a small portion of the left head, then a small portion of the right head, etc. The theoretically important point here is that, during copying, attention is allocated initially exclusively to one of the heads, and subsequently, exclusively to the other. Therefore, in neglect it is likely that the left side of each figure would be neglected.

A strikingly different situation arises in the Tracing Condition. During tracing, a line must be drawn over the outline representing the stimulus image that is already present on the page. During tracing, the tracer is not required to engage in a series of successive, discrete, iterative, episodes of reproduction of a portion of the stimulus, as occurs in copying. Instead, tracing occurs via a more fluid, single, continuous process, operationalized over the stimulus as a whole. During tracing, the tracer maintains the pen’s pursuit of the stimulus outline from the starting point until the trace is completed, that is, until the perceived stimulus has been reproduced in its perceived entirety. This means that it is likely that the left side of that whole perceived stimulus may not be attended to in neglect. In contrast, during copying of the stimulus, the separate figures may be represented as two distinct units and therefore the left side of each of those units may not be attended to in neglect.

**Figure 2:**
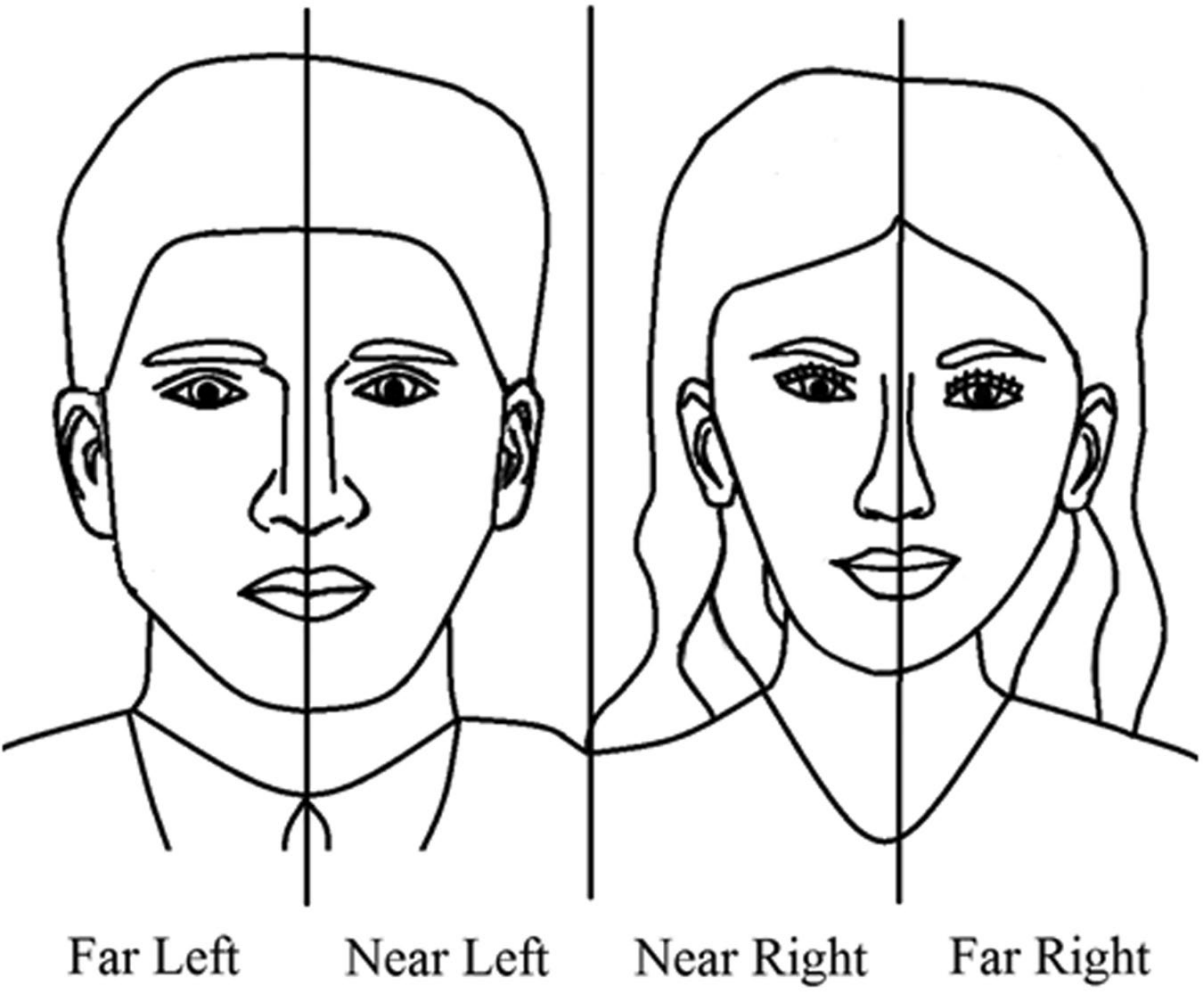
Regions of interest on one of the stimuli used in the Tracing and Copying Conditions. The stimulus was divided into equal quadrants that provided four regions of interest along the horizontal plane (Far Left, Near Left, Near Right and Far Right) for the behavioral (figure completion accuracy) and eye movement measures (fixations).

It is our contention that differences in symptoms of neglect arise due to implicit task requirements under conditions of a constant external visual environment, and that different symptoms may arise in the same patient, reflecting changes in the nature of visuo-cognitive processing in response to specific task demands. To be clear, we do not regard egocentric and allocentric presentations of neglect to necessarily reflect categories of neglect patients (NPs) that have distinct disorders of attentional processing. To allow us to quantify attentional allocation across the stimulus, we divided the stimulus array into four regions (Far Left, FL; Near Left, NL; Near Right, NR; and Far Right, FR; see Fig. 2). For copying, we expected that NPs would allocate their attention sequentially, first exclusively to the figure on the right, and then exclusively to the figure on the left. For this reason, in relation to copying accuracy (i.e., percentage completion of the figure), we predicted that NPs would fail to produce the left side of each figure in turn (i.e., reduced completions in the FL and NR regions), relative to matched control participants. Conversely, during the Tracing Condition, we anticipated that NPs would allocate their attention to the stimulus as a whole, and therefore, have reduced completion accuracy for the left portion of the whole array, that is, the entire left figure (FL and NL regions). Furthermore, on the assumption that eye movement behaviour would reflect such attentional allocation, in line with our completion predictions, we also anticipated that NPs would spend more time fixating the NR and FR regions than the NL and FL regions during tracing, and more time fixating the NL and FR regions than the FL and NR regions during copying. In matched control participants, we expected the time spent fixating each of the regions to be equivalent.

## Results and Discussion

In line with our predictions, in the Tracing Condition, NPs had reduced completion accuracy (proportion of component parts of the image being successfully reproduced) for the figure presented on the left side of the stimulus compared to the control participants (Stroke Controls [SCs] and Older Adult Controls [OACs]) demonstrating a pattern of behaviour consistent with egocentric neglect (see individual patient data for figure completion accuracy in Table S1 and the pictures patients produced in the Supplementary Information). In the Copying Condition, an allocentric pattern of neglect was apparent, with NPs having reduced completion accuracy for the left side of each figure (FL and NR regions). That is, in the Copying Condition, NPs demonstrated increased accuracy (copying more parts of the image) in the NL region (the right side of the left face) compared to in the Tracing Condition. This indicates that the task demands resulted in more attention being allocated to the left face in the Copying compared to the Tracing Condition.

**Figure 3:**
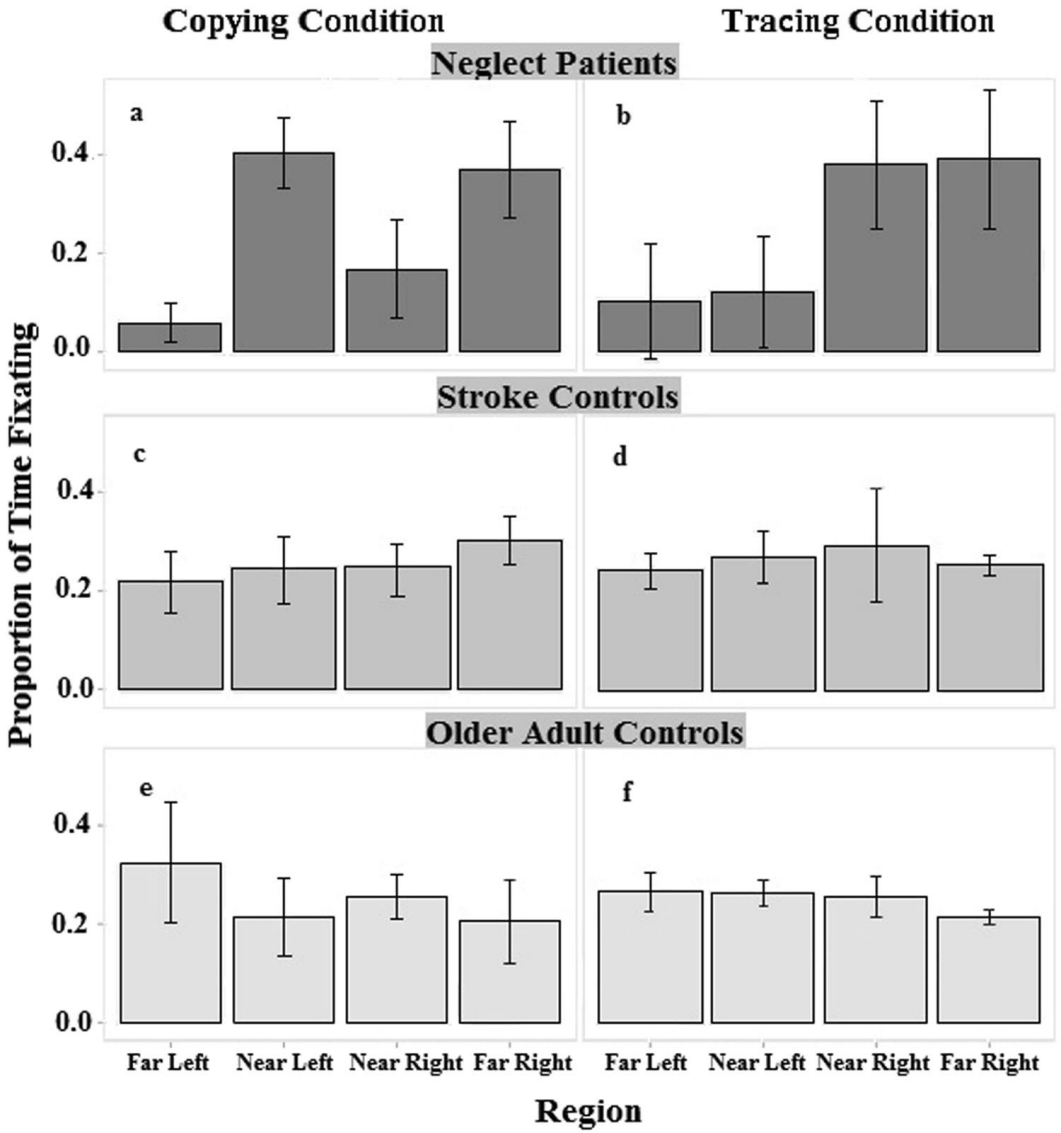
Proportion of the total trial time spent fixating the four regions of the stimulus (Far Left, Near Left, Near Right and Far Right) during the Tracing and Copying Condition for the Neglect Patients (NPs), Older Adult Controls (OACs) and Stroke Controls (SCs). Error bars represent the 95% confidence interval around the mean. The same pattern of behaviour was found for the number of gazes meaure (see Fig. S2 in the Supplementary Information).

To examine this further, visual sampling of the information was analysed by comparing the proportion of time spent fixating different regions across the stimulus. We used the proportion of time spent fixating, rather than the actual time spent fixating to counteract differences in completion times across participants and tasks. There were significant interactions between region and group, *F*(6, 51) = 10.51, *p* < 0.001, *η*_*p*_^*2*^ = 0.55, task and region, *F*(3, 51) = 4.62, *p* = 0.006, *η*_*p*_^*2*^ = 0.21, and region, group and task, *F*(6, 51) = 6.08, *p* < 0.001, *η*_*p*_^*2*^ = 0.42. These interactions arose from NPs differing in the proportion of time spent fixating regions in the stimuli across the two tasks, with a significant main effect of region, *F*(3, 15) = 13.09, *p* < 0.001, *η*_*p*_^*2*^ = 0.72, and region by task interaction for NPs, *F*(3, 15) = 8.24, *p* = 0.002, *η*_*p*_^*2*^ = 0.62, which was absent for SCs, *F*(3, 12) = 0.69, *p* = 0.502, and OACs, *F*(3, 24) = 1.75, *p* = 0.184. As with the completion data, the NPs spent less time fixating the left side of the whole stimulus (the FL and NL regions) in the Tracing Condition than the right side of the stimulus, but less time fixating the left side of each figure (FL and NR) than the right sides of the figures (NL and FR) in the Copying Condition. This pattern is apparent in the data presented in Fig. 3.

As predicted, during the Tracing Condition, NPs neglected information on the left of the entire image, suggesting an egocentric frame of reference was operating during that task. Information to the left of the patients’ midline was fixated for less time compared to regions to the right of their midline, *t* (11) = 4.08, *p* = 0.002. This suggests that the NPs were representing the stimulus as a whole and, therefore, neglecting the left side of the stimulus.

In stark contrast, when completing the Copying Condition, an allocentric frame of reference appeared to be operating, with NPs spending less time fixating the left side of each figure (corresponding to the far left [FL] and near right [NR] regions of the stimulus) than the right side of each figure (corresponding to the near left [NL] and far right [FR] regions of the stimulus). In the Copying Condition, NPs spent less time fixating the FL and NR regions (the left side of both of the figures) than the NL and FR regions (the right side of the figures), *t* (11) = 6.07, *p* < 0.001. This pattern was also evident in the number of fixations made by NPs in the regions for the two different tasks (see Fig. S2 in the Supplementary Information).

**Figure 4:**
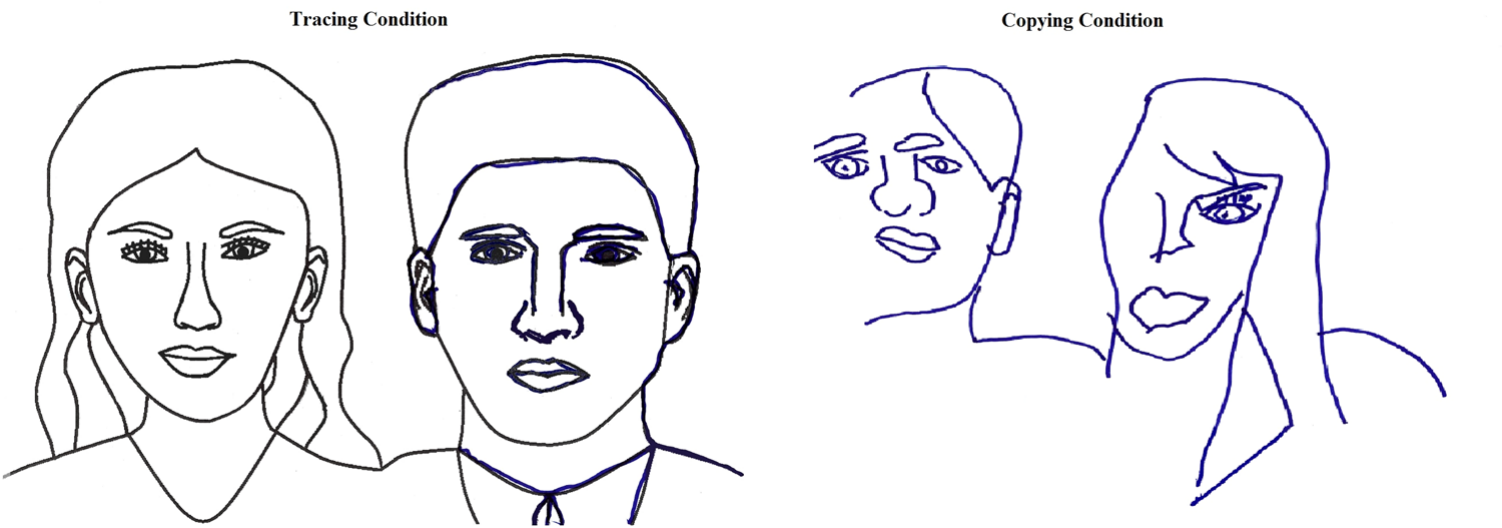
Example of an NP’s performance (Case 1) in the Tracing and Copying Conditions. The transparent regions on the Tracing Condition figure are those areas that the NP failed to draw over and the lines indicated in blue are those that were traced. For the Tracing Condition the NP neglected the whole left face (FL and NL regions) and parts of the left side of the right face (NR region) demonstrating egocentric neglect. Allocentric neglect was demonstrated by the same NP during the Copying Condition, where the left sides of the left (FL region) and right faces (NR region) were now neglected.

To be clear, the task demands (whether the participant was required to trace or copy an image) had an impact on how the task was undertaken, affecting how the stimulus was represented and, subsequently, which information was neglected. In the Tracing Condition, where the NPs were required to trace over the whole image, the eye movements provided evidence to indicate that they were representing the stimulus as a whole, which was reflected in a failure to sample the whole left figure (in the contralesional regions of the stimulus [FL and NL]; see example in Fig. 4). However, when NPs had to form and store a representation in order to accurately reproduce the figure in the Copying Condition, NPs appeared to be operating on one figure at a time, resulting in the left side of each figure being fixated for less time compared to the right side of the figure. Therefore, processing of the two figures appeared to be serial and sequential in the Copying Condition as opposed to parallel in the Tracing Condition.

Importantly, the overriding pattern of performance across the group of NPs was one consistent with allocentric neglect in the Copying Condition and egocentric neglect in the Tracing Condition (see Table S1 in the Supplementary Information and the images produced by the patients). This consistency across all NP participants suggests that it was not the ‘type’ of neglect that the patient presented with (e.g., egocentric or allocentric) that resulted in the pattern of behaviour observed, but instead, it was the particular demands in terms of attentional allocation for the different tasks, that caused differential processing of information, and that is what resulted in particular spatial information being neglected in the different tasks. The nature of neglect symptoms, therefore, can arise as a direct consequence of what the patient is required to do during a given task, and how they represent the stimulus based on the specific task requirements. The fact that all NPs presented with both ‘types’ of neglect symptoms under different circumstances raises questions in relation to procedures associated with NP categorisation, and also demonstrates the possibility that egocentric and allocentric neglect may neither be mutually exclusive, nor necessarily distinct syndromes. We have demonstrated that an egocentric pattern of neglect is exhibited during one experimental condition, and when interacting with the same stimulus in a different experimental condition, an allocentric pattern of neglect is apparent within the same patients, when the lesion location remains constant.

The findings demonstrate that allocentric neglect can be exhibited if the task encourages each object to be processed individually, as in the Copying Condition. Therefore, this type of neglect as demonstrated in some studies may reflect the task demands imposed on the participant, rather than particular deficits experienced by different NPs. Different task demands resulting in differential patterns of neglect may be able to explain a number of findings for the existence of allocentric neglect. These findings imply that there are different levels of granularity for representation of the visual environment (e.g., all space in front of the participant, the piece of paper presented to them, images presented on the page). Depending on the representation that is activated as a result of the requirements of the task, differential information will be neglected. Although these findings do not preclude the possibility of different NPs’ presenting with different types of neglect (e.g., egocentric and allocentric), they imply that the nature of the neglect symptoms that manifest may reflect the demands of the task that is being conducted by the NP at the current moment in time. To be clear, the task demands (whether the participant was required to trace or copy an image) had an impact on how the task was undertaken, affecting how the stimulus was represented and, consequently, which information was neglected.

Another important implication of these findings is that they demonstrate that an area of space that is not attended to in one condition can be voluntarily attended to in another condition by a HN patient, without instruction to do so. For example, HN patients spent more time fixating a region of space that was neglected, the NL region (as this was the right side of the left face), in the Copying Condition compared to in the Tracing Condition. Thus, the results demonstrate that regions of neglect may be moderated through manipulation of task demands that require operationalization of different frames of reference. On this basis, it seems reasonable to conclude that neglect is a dynamic disorder in nature^23^, and that task demands can have a direct impact on the presentation of neglect: specifically the reference frame that operates at any given time. It is important to note that this is the first demonstration of egocentric and allocentric neglect in the same group of NPs for the same stimulus that has not been modified to influence processing.

In summary, all NPs in the study exhibited different ‘types’ of neglect under different circumstances (for different task requirements), indicating that the processing and representation of the stimulus differed depending on the current task being undertaken. Therefore, activation of egocentric representations (or object-based allocentric representations) may be determined by the requirements of the task in which the NP is currently engaged. Differences in symptoms of neglect can arise due to implicit task requirements under conditions of a constant external visual environment, and those different symptoms may arise in the same patient for whom lesion location remains constant. Furthermore, such differences reflect changes in the nature of visuo-cognitive processing in response to specific task demands. To be clear, task demands (i.e., tracing or copying an image) had a direct impact on how the task was undertaken, affecting how the stimulus was represented, and subsequently, which information was neglected. More generally, our findings provide important insight into factors affecting neglect symptoms that are critical for patient diagnosis.

## Method

### Participants

The sample comprised 6 stroke patients who had right hemisphere damage with left hemispatial neglect (NPs), 5 stroke patients who had had a right hemisphere stroke but did not exhibit neglect (Stroke Controls; SCs) and 9 healthy older adult controls (Older Adult Controls; OACs). There were 15 females and 5 males in total. All were right handed. NPs had an age range of 50–78 years (*M* = 64.67 years, *SD* = 11.31 years), SCs 62–84 years (*M* = 73.20 years, *SD* = 9.83 years) and OACs 52–82 years (*M* = 67.56 years, *SD* = 9.95 years). All NPs were in the acute phase (prior to 3-months post-stroke), with the number of days post-stroke at the time of participating in the experiment ranging from 2–59 (*M* = 25.00, *SD* = 19.60) for NPs and 2–100 for SCs (*M = *47.00, *SD* = 42.83), which did not differ significantly, *t*(9) = −1.133, *p* = 0.143. NPs and SCs did not differ in number of years spent in education, *t* (9) = 0.31, *p* = 0.761. However, both NPs and SCs spent fewer years in education than the OACs, *t* (13) = 2.47, *p* = 0.028, although this was marginal for SCs, *t* (12) = 1.90, *p* = 0.081. The presence or absence of neglect was quantified from the performance on a sub-set of the BIT^24^. Using the clinical diagnosis, the BIT score and observation of behaviors during the tasks indicative of neglect (e.g. immediately orienting to the right and repeatedly crossing through ipsilesional targets) the presence of neglect was confirmed (see Table 1). Patients all consistently exhibited classic egocentric patterns of neglect as indexed by these standardised tasks (the BIT tasks).

**Table 1:** Number of Days Post-Stroke, BIT score, Aetiology, Lesion Area and Visual Field Defects for the Neglect Patients (NPs) and Stroke Controls (SCs).

Participant Number	Group	Days Post-Stroke	BIT score	Aetiology	Lesion Location	Visual Field
Case 1	NP	59	81.05	Infarct	R MCA territory	LH
Case 2	NP	28	67.28	Infarct	R MCA territory; R TACs	LLQ
Case 3	NP	25	83.36	Infarct	R MCA territory	None
Case 4	NP	26	67.90	Infarct	M1 segment of the R MCA extending to the M2 insula branches; effacement of the convexity sulci and R sylvian fissure	None
Case 5	NP	2	66.67	Infarct	R parietal and lateral occipital lobe	None
Case 6	NP	10	87.04	Infarct	R MCA territory; R frontoparietal, frontal operculum and insula	None
SC 1	SC	18	100.00	Infarct	R caudate nucleas; R lacunar	None
SC 2	SC	25	98.15	Infarct	Basal ganglia; R globus pallidus/putamen; R lacunar	None
SC 3	SC	100	96.10	Infarct	R precentral gyrus	None
SC 4	SC	6	96.30	Infarct	R lacunar	None
SC 5	SC	86	98.77	Infarct	MCA territory; insula cortex; R PACs	None

NP = Neglect Patient; SC = Stroke Control; R = Right; MCA = Middle Cerebral Artery; TACs = Total Anterior Circulation infarct; LH = Left Hemianopia, LLQ = Lower Left Quadrantanopia.

Patients were recruited consecutively during the course of the project. The inclusion criteria consisted of the following: the patient had a stroke and had been diagnosed with hemispatial neglect, was above the age of 18 and was capable of making an informed decision of whether to partake in the research (based on consultant psychologist’s/stroke team’s assessments). The exclusion criteria were as follows: diagnosis of dementia, extreme motoric dysfunction (e.g. unable to make and sustain arm movements on the side unaffected by the stoke), and/or partial sightedness due to damage/degradation of the structure of the eye/retina.

Lesion location information was obtained from the available Computed Tomography (CT) head/brain scan reports contained within the patients’ medical notes at the admitting hospital. The majority of the NPs had a Partial Anterior Circulation (PACs) or Total Anterior Circulation (TACs) unilateral infarct within the Right Middle Cerebral Artery (RMCA) territory lesioning the right frontoparietal or occipital lobes and for some patients including the right insula, sulci, sylvian fissure, operculum, the internal capsule, and lentiform nucleus. One NP (Case 5) also had damage to the lateral aspect of the right occipital lobe but, note, that this participant did not exhibit visual field loss. The SCs tended to have RMCA initiated infarcts resulting in damage to the right caudate nucleus, insula, putamen, lacunar, precentral gyrus and basal ganglia.

All experiments were performed in accordance with the Declaration of Helsinki. Informed consent was gained from each participant. This study received School of Psychology, University of Southampton and NHS Southampton REC National Research Ethics Service ethical approval and was adopted by the UK Stroke Research Network (SRN) portfolio. The patients were recruited from the following sites included in the project: Southampton General Hospital, Royal Hampshire County Hospital, Royal Bournemouth Hospital, Poole General Hospital and Lymington New Forest Hospital.

### Stimuli

In the Tracing Condition, participants were presented with a line drawing of two faces displayed on an A4 landscape piece of paper (see Fig. 1). Participants were required to mark all the lines within the image with a pen as if tracing the image onto another piece of paper on top of the original.

The Copying Condition required the participant to copy the line drawing of two faces, similar to those included in the Tracing Condition onto a separate piece of paper placed beneath the original. There were four versions of this stimulus to be used in these conditions (see Fig. 1). Participants either received figures labelled 1 and 4 or 2 and 3 in Fig. 1). These were counterbalanced across participants for the condition in which the stimulus appeared (i.e. Tracing or Copying Condition) and the conditions were counterbalanced for order presentation across participants.

### Screening Tests

A number of tests were conducted prior to the tasks being completed and information was obtained to acquire measures of pre-stroke IQ (NART), memory (MMSE), visual acuity (Snellen), visual fields (Confrontation Visual Field Testing), and inattention (BIT).

Demographic information such as age, handedness, gender and lesion location for patients was also gathered. The NPs did not significantly differ on the MMSE, NART and Snellen Visual Acuity Test from SCs, *t* (9) = 2.03, *p* = 0.087, *t*(9) = 1.50, *p* = 0.169, *t*(9) = 0.01, *p* = 0.989. These screening results demonstrate that any poor task performance limited to NPs would be unlikely to be a result of differences between NPs and controls on IQ, memory or visual acuity measures. As expected, groups significantly differed on the BIT, *F* (2, 17) = 42.47, *p* < 0.001, η_p_^2^ = 0.83. NPs had far poorer performance than SCs, *t* (9) = 5.79, *p* = 0.002, and OACs, *t* (13) = 6.21, *p* = 0.001, demonstrating clinical neglect. Thus, any differences in figure completion accuracy or patterns of eye movement measures between NPs and control participants are likely to reflect inattention in neglect.

### Apparatus

A 76.2 cm × 59.9 cm easel positioned at a slant of 67° was used to display the stimuli. This was placed on a desk in front of the participant and was located at an average viewing distance of 55 cm. The participants were seated in an adjustable chair with their heads resting on a chin rest in order to minimise head movements and to ensure the stimulus was centred on the sagittal mid-plane of the participants’ head and trunk.

The eye-tracking data were gathered using an Applied Science Laboratories E5000 eye-tracker, running at 60 Hz. This video-based infrared tracker monitored fixation position by tracking the centre of the pupil, as well as the first-surface corneal reflection. The eye-tracking video cameras were mounted onto a set of spectacle frames (acquisition device), which were worn by the participants during the experiment. Two cameras, the eye camera and the scene camera provided information about eye position and the scene in front of the participant. The eye camera was directed towards the right eye and recorded the movements made by the eye (including the pupil and corneal reflection). The corneal reflection was illuminated via a light-emitting diode (LED) located underneath the eye camera. Participants viewed the scene binocularly but only the movement of the right eye was recorded. The scene camera recorded the scene in front of the participant as the trial progressed. The two video feeds were recorded by a JVC GR-DF4SOV camcorder operating at 30 frames per second, housed in a backpack. The backpack also contained a power supply for the camera and a multiplexer for the eye-tracking equipment.

### Design

This was a mixed design with three variables. The first between participants variable was ‘group’, with three levels (NPs, SCs, OACs). The task completed was a within participants variable, with participants being assessed on both the Tracing and Copying Conditions whilst their eye movements were recorded. Region of interest for the eye movement measures was a further within participant variable, with the stimulus being divided into four regions (FL, NL, NR and FR, see Fig. 2).

### Procedure

The stroke patients (NPs and SCs) took part in the study individually in a well-lit, quiet room either within the admitting hospital, or within their own homes if they had been discharged. The OACs were all tested in the same environment in the School of Psychology at the University of Southampton. The first stage involved completion of the screening tests and obtaining of any additional information. Once this stage was completed, the participants were then given a break before the experiment commenced. On some occasions, due to chronic fatigue that stroke patients often experience, the experiment was conducted on a separate occasion (usually the next day). If the experiment was not conducted within a week of screening, the participants were reassessed and those are the measures that are reported in this section.

Participants were seated in a chair (or a wheelchair if required) in front of a desk on which the easel was placed. Participants wore the head-mounted eye tracker acquisition device like a pair of spectacles. When the experimenter was satisfied that the positioning of the cameras would obtain high-quality recordings of the eye movements and the scene, recording of the video feeds commenced.

Calibration procedures prior to the experiment required participants to follow a laser-pointer that guided the participants to look directly at the centre of five points presented in the scene (subtending approximately 2° × 2° of visual angle) sequentially. One calibration point was placed at each of the corners, and at the centre, of an A4 piece of paper, which was located at the centre of the easel. A stimulus (the line, star or letter cancellation task) was then placed onto the centre of the easel. If required, the participant was able to take a break following completion of one task, after which another calibration was undertaken before the next task was administered.

For the Tracing Condition, participants were instructed to draw over the lines of the image in the stimulus with a marker pen, as if they were tracing the image onto a piece of paper presented over the top of the stimulus. For the Copying Condition, participants were required to copy the image onto an A4 piece of paper presented beneath the stimulus. Participants in both conditions were asked to indicate when they had finished the task by placing the pen on the table and looking at it. No time limit was imposed to ensure that limited time was not a factor contributing to incomplete figure tracing or copying.

### Data Analysis

Using specialised software developed within the Psychology Academic Unit, the fixation position of the eye in the scene (obtained from the eye camera) was superimposed onto the scene footage (obtained from the scene camera). This was achieved by calibrating the position of the eye at each of the five calibration points that were present in the scene footage during the calibration procedure. A verification procedure (a second calibration) was included to ensure that the fixation cross was in the correct position within the scene following calibration (i.e. that the fixation cross fell on each of the calibration points during the second procedure). If this was not achieved, calibration was attempted again and if it was not possible to accurately calibrate the data, the trial was excluded from the analyses.

An.avi file was saved incorporating the point of fixation within the scene for each frame in the video (approximately 2,000 frames per trial). Using video analysis software (VirtualDub) to step through the.avi footage frame-by-frame, the position of the fixation with respect to regions of interest imposed on the stimuli (described in the next section) was hand-scored for each frame. Frames were scored for the period of the trial in which the task was being completed (i.e. not for eye movements made during instruction to the participants). When the fixation cross was not available in a frame due to an eye-blink or tracker loss, these frames were not included in the data. The temporal accuracy was determined by the rate of recording on the camcorder, which was one frame every 33 ms. All of the trials were hand-scored by two individuals in order to achieve scoring reliability. The eye movement measures (proportion of time spent fixating each region, number of gazes) were extracted from the raw data via algorithms developed in RStudio^TM^. A gaze was defined as commencing when a saccade was made into a region. The end of the gaze occurred when the eye transgressed a region boundary, i.e. made a saccade to another region. Proportion of trial time spent fixating each region is the amount of time the participant spent fixating that region as a function of the total time spent completing the trial.

### Data Availability

The datasets generated during and/or analysed during the current study are available from the corresponding author on reasonable request.

## Electronic supplementary material

Supplementary Information
