# Supplementary material for: Neglect Patients Exhibit Egocentric or Allocentric Neglect for the Same Stimulus Contingent upon Task Demands
Source: Sci Rep. 2017 May 16;7:1941. doi: 10.1038/s41598-017-02047-x (PMC5434059; doi:10.1038/s41598-017-02047-x)

Leyland, Godwin, Benson, & Liversedge

Neglect Patients Exhibit Egocentric or Allocentric Neglect for the Same Stimulus Contingent upon Task Demands

Supplementary Information

Figure Completion Accuracy

The accuracy of the figure tracing and copying for each participant was scored using the following method: for each region (left figure vs. right figure) a score out of 10 was available, with one point being deducted for each part of the figure that was missing or incomplete. One point was deducted for each of the following features that were incomplete or missing in each region: top hairline, bottom hairline, eye brow, eye (including pupil), nose, ear/side hairline, mouth, neck/chin, shoulder, collar/neckline (see Figure S1 for these sections highlighted on the figures included in these conditions). There were a maximum of ten marks per region for figure encoding or copying accuracy; 40 marks overall. This was transformed to proportion accuracy for each region.

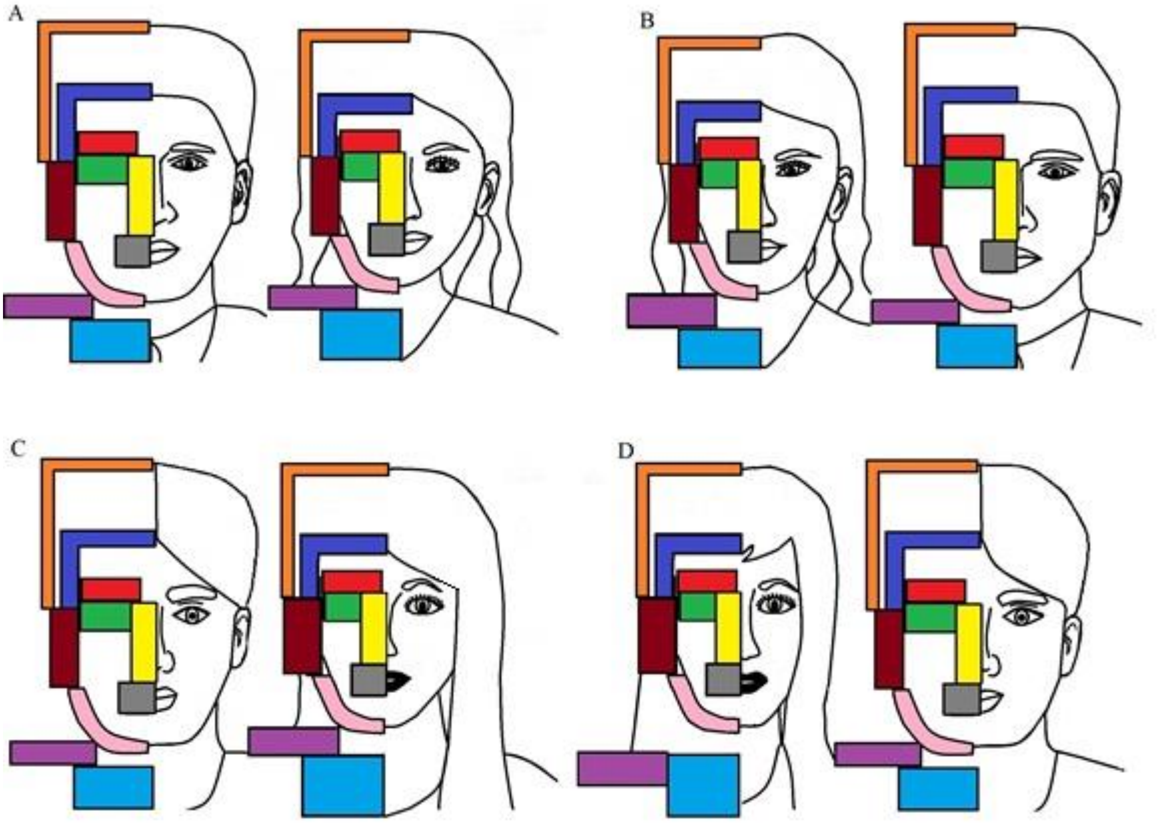

Figure S1. The regions that were used in the computation of figure completion accuracy for each of the four stimuli are denoted by the different coloured boxes.

Table S1

Figure completion accuracy in each of the regions of interest on the stimulus (Far Left; FL, Near Left; NL, Near Right; NR, Far Right; FR) for each Neglect Patient during the Tracing and Copying Conditions. This measure reflects the behavioural accuracy in completing elements of the stimulus in the trace/copy produced.

**Case Number**	**Target Completion Accuracy in the Tracing Condition**				**Target Completion Accuracy in the Copying Condition**			
	**FL**	**NL**	**NR**	**FR**	**FL**	**NL**	**NR**	**FR**
**Case 1**	0%	0%	60%	90%	40%	100%	30%	100%
**Case 2**	50%	50%	80%	100%	80%	90%	100%	100%
**Case 3**	0%	0%	60%	100%	80%	100%	80%	90%
**Case 4**	0%	0%	80%	100%	20%	80%	60%	80%
**Case 5**	50%	80%	60%	70%	50%	80%	80%	80%
**Case 6**	90%	90%	100%	100%	40%	80%	50%	70%
**Mean (SD)**	32%	37%	73%	93%	52%	88%	67%	87%
	(37%)	(42%)	(16%)	(12%)	(24%)	(9%)	(25%)	(12%)

Number of Gazes in Each Region

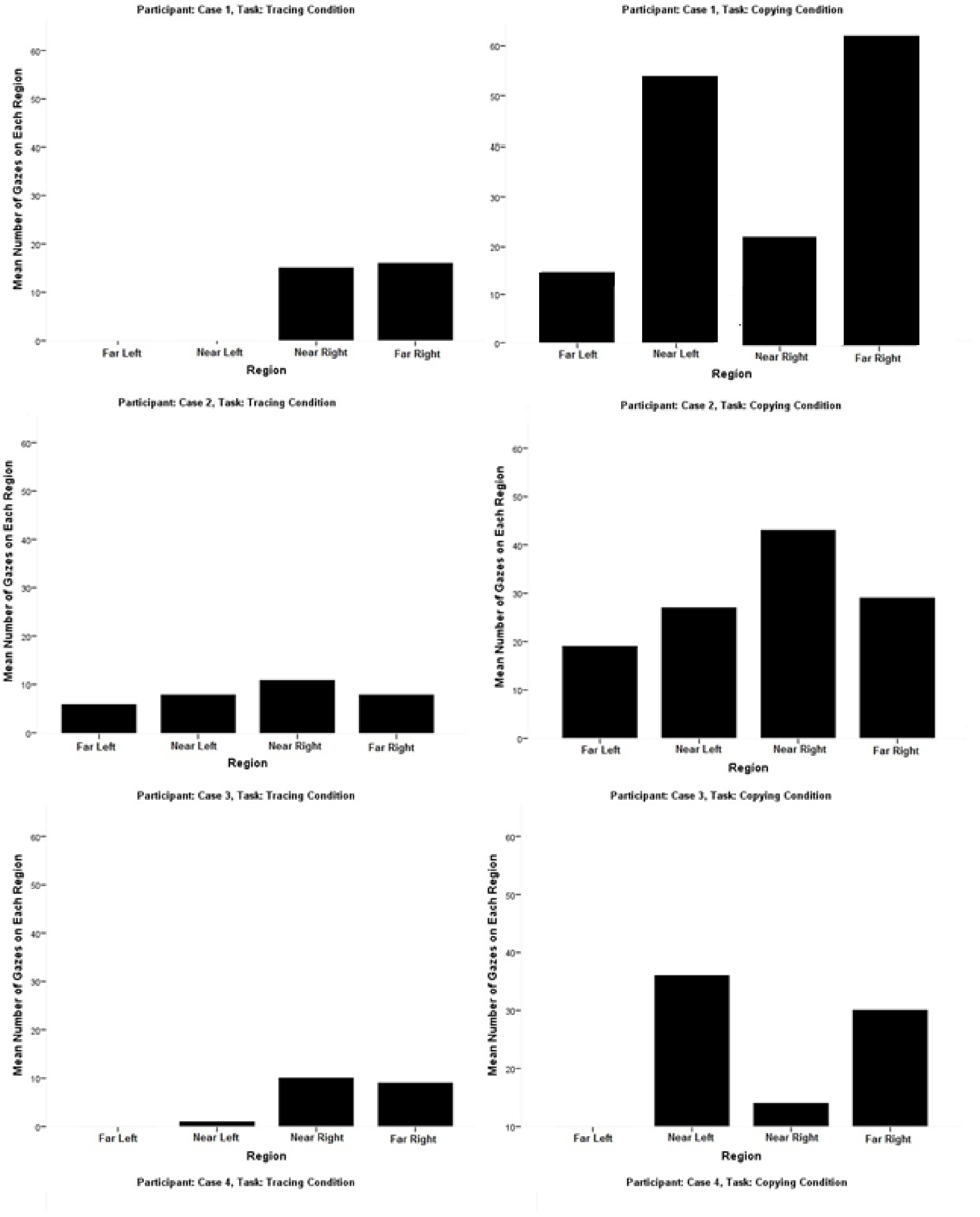

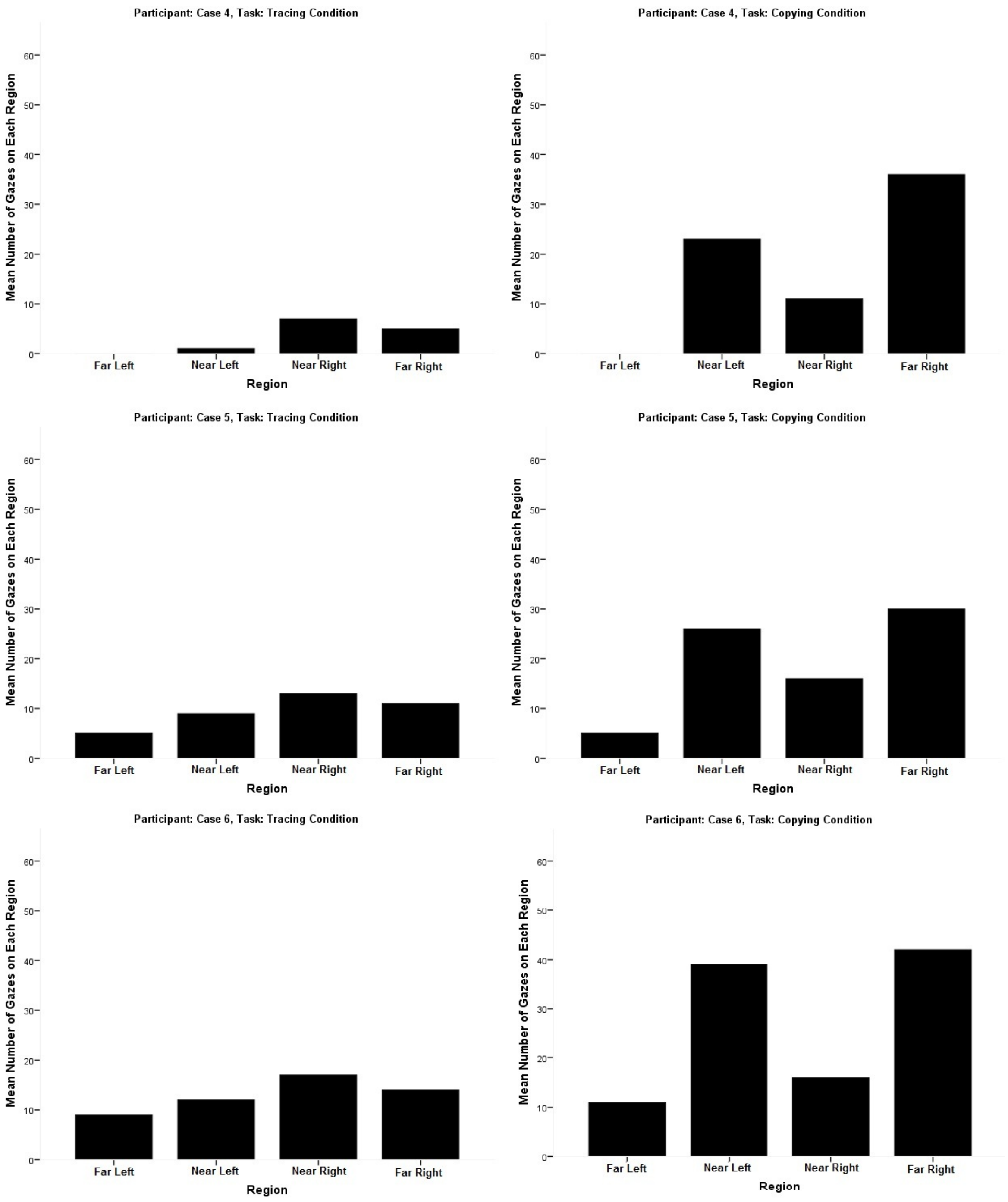

Figure S2. The number of gazes measure reflects the number of occasions a region was visited (a gaze was defined as a saccade being made to a region; a new gaze commencing when the eyes transgressed a region boundary, i.e., a saccade was made to another region). These graphs demonstrate the same pattern as the proportion of time spent fixating each region measure, with fewer gazes being made to the left side of the stimulus during the Tracing Condition, indicating egocentric neglect. However, allocentric processing was indicated by fewer gazes being made to the left side of each face in the stimulus during the Copying Condition.

Case 1 Copying Condition

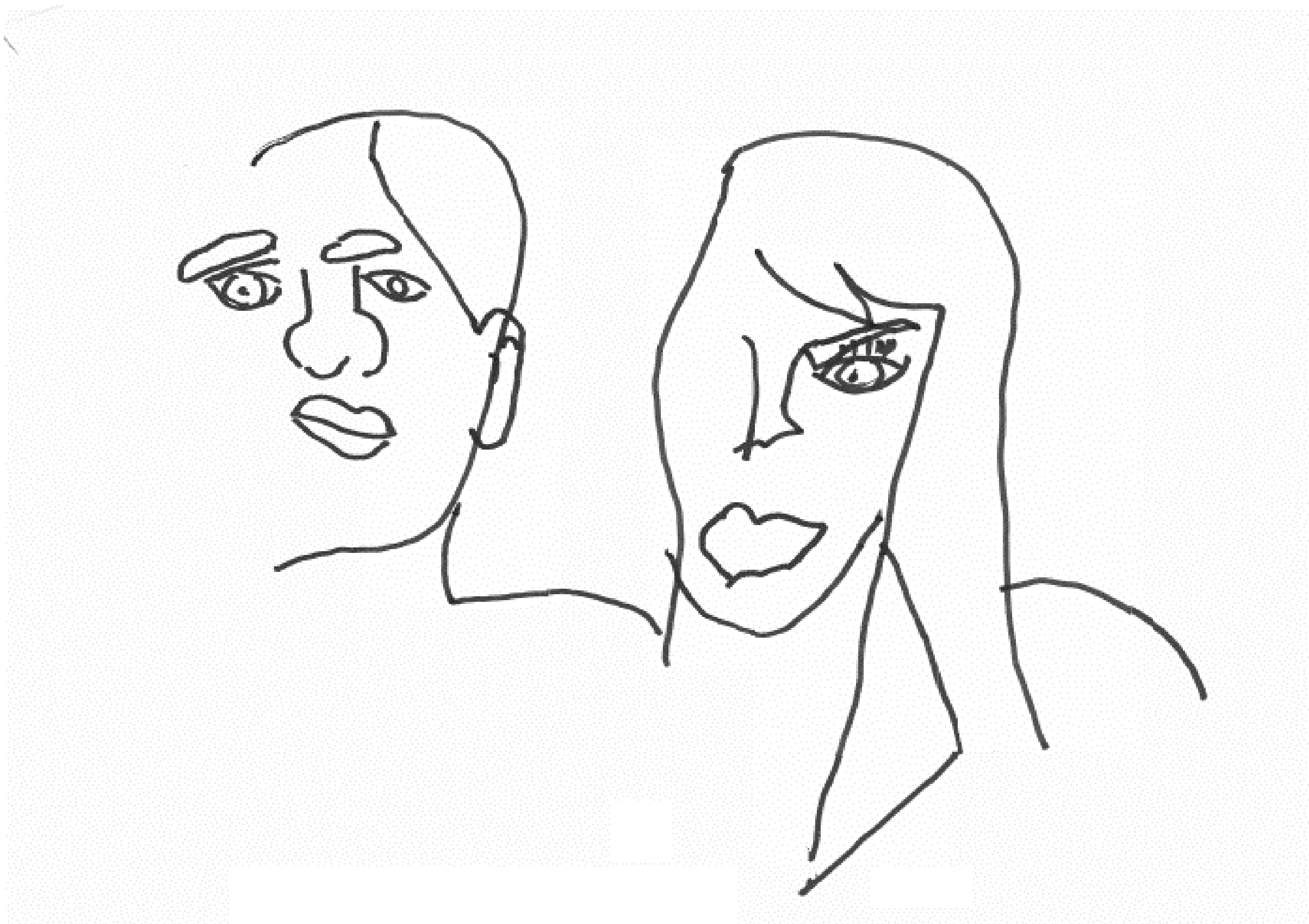

Case 1 Tracing Condition

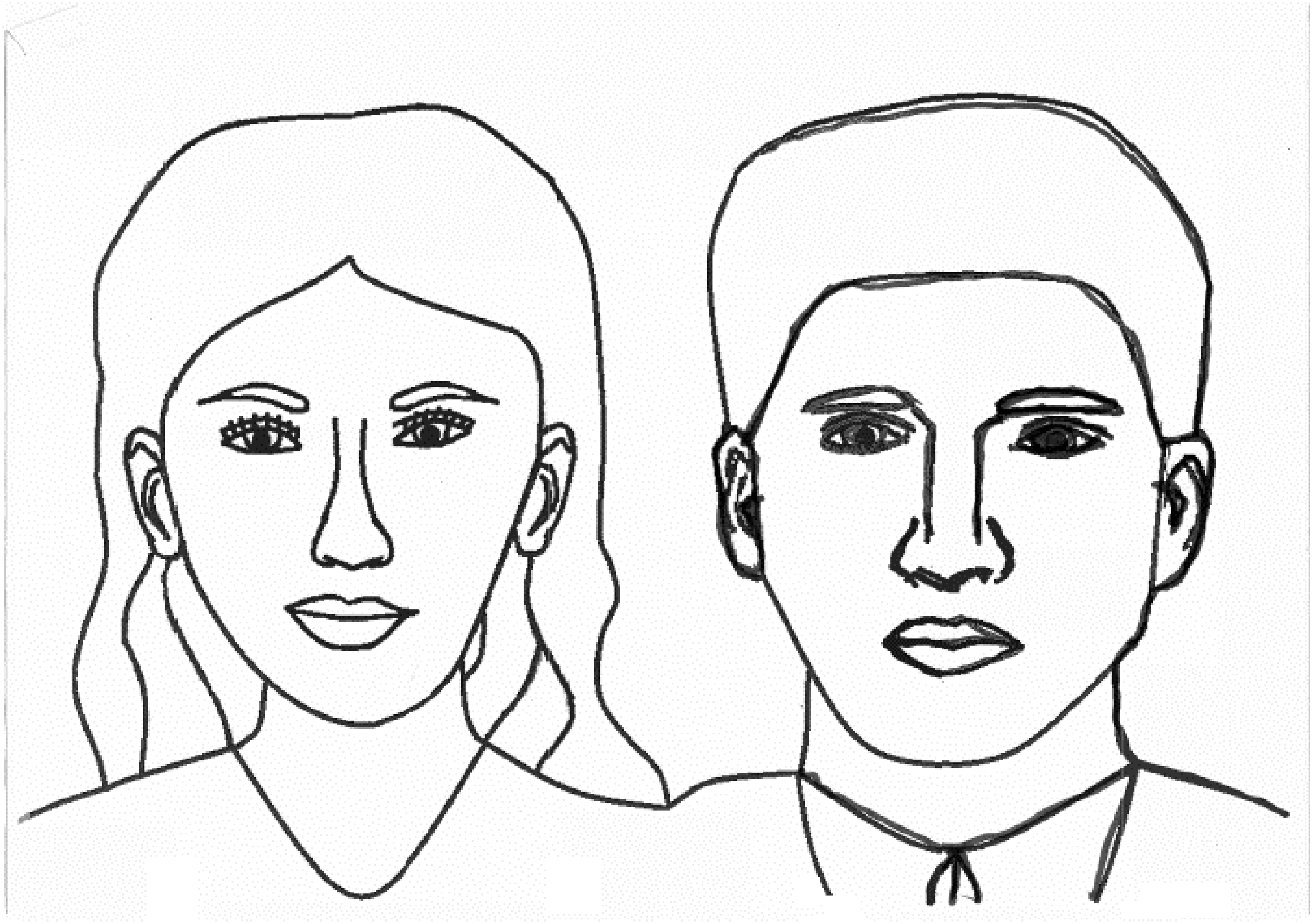

Case 2 Copying Condition

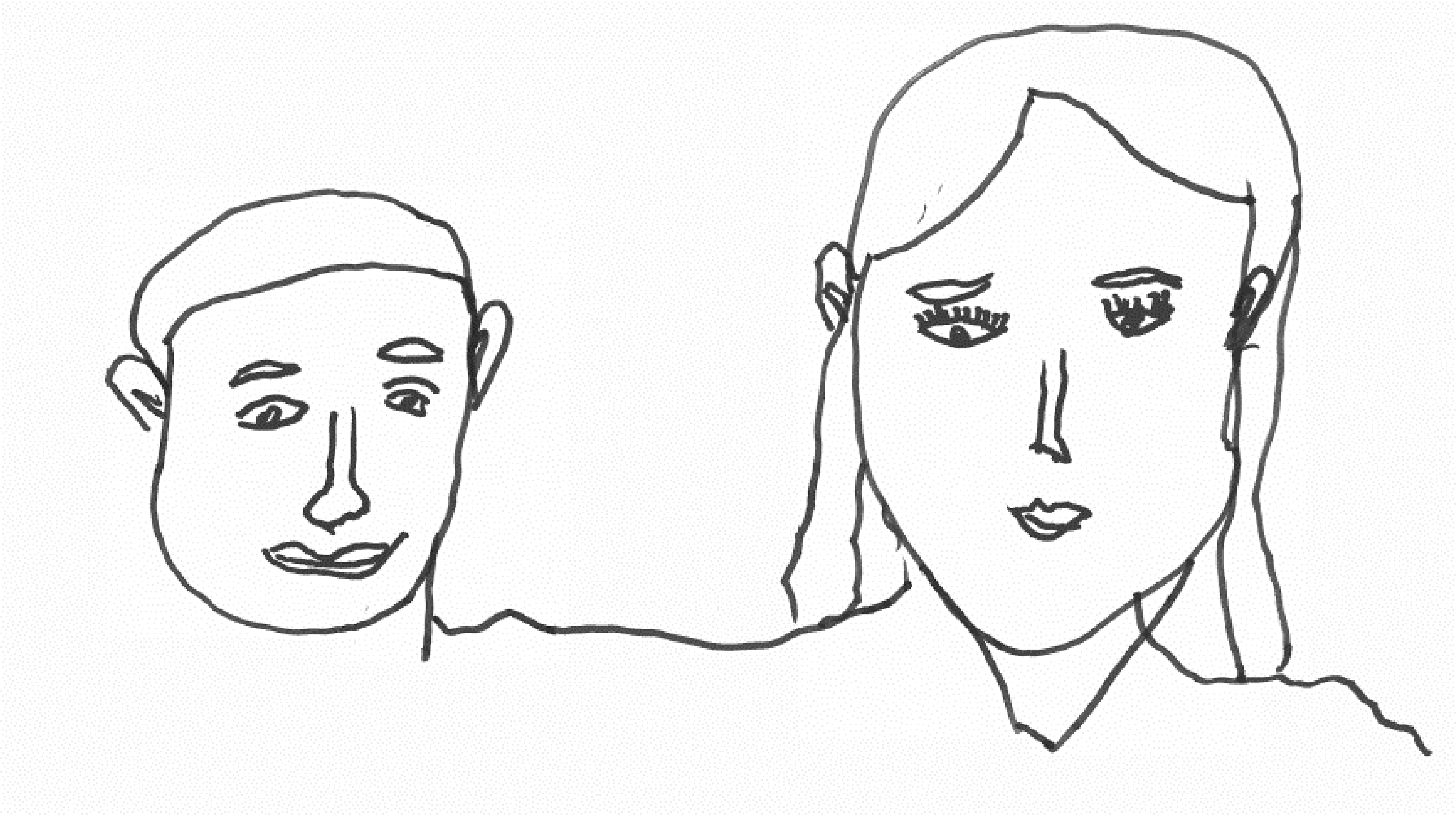

Case 2 Tracing Condition

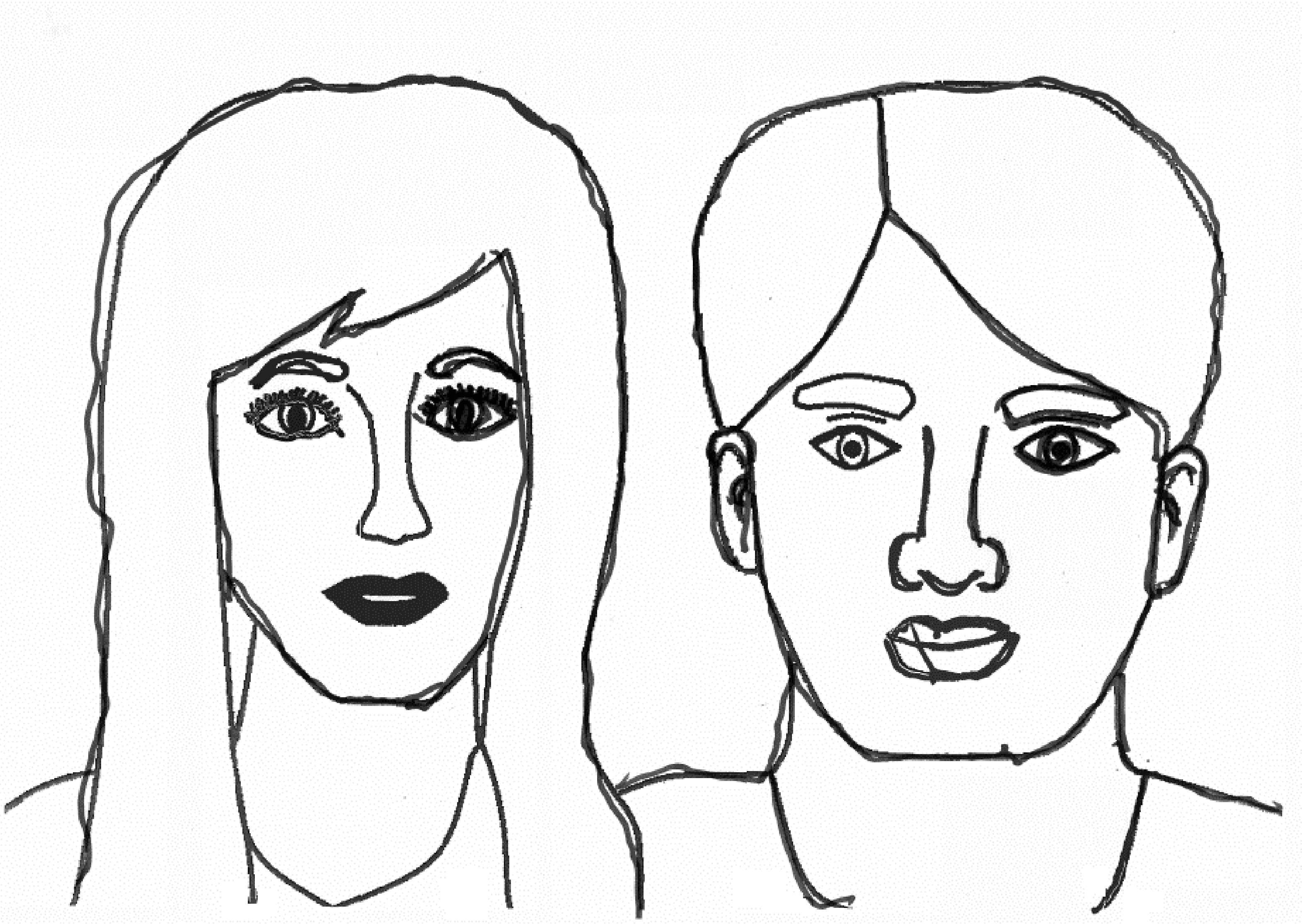

Case 3 Copying Condition

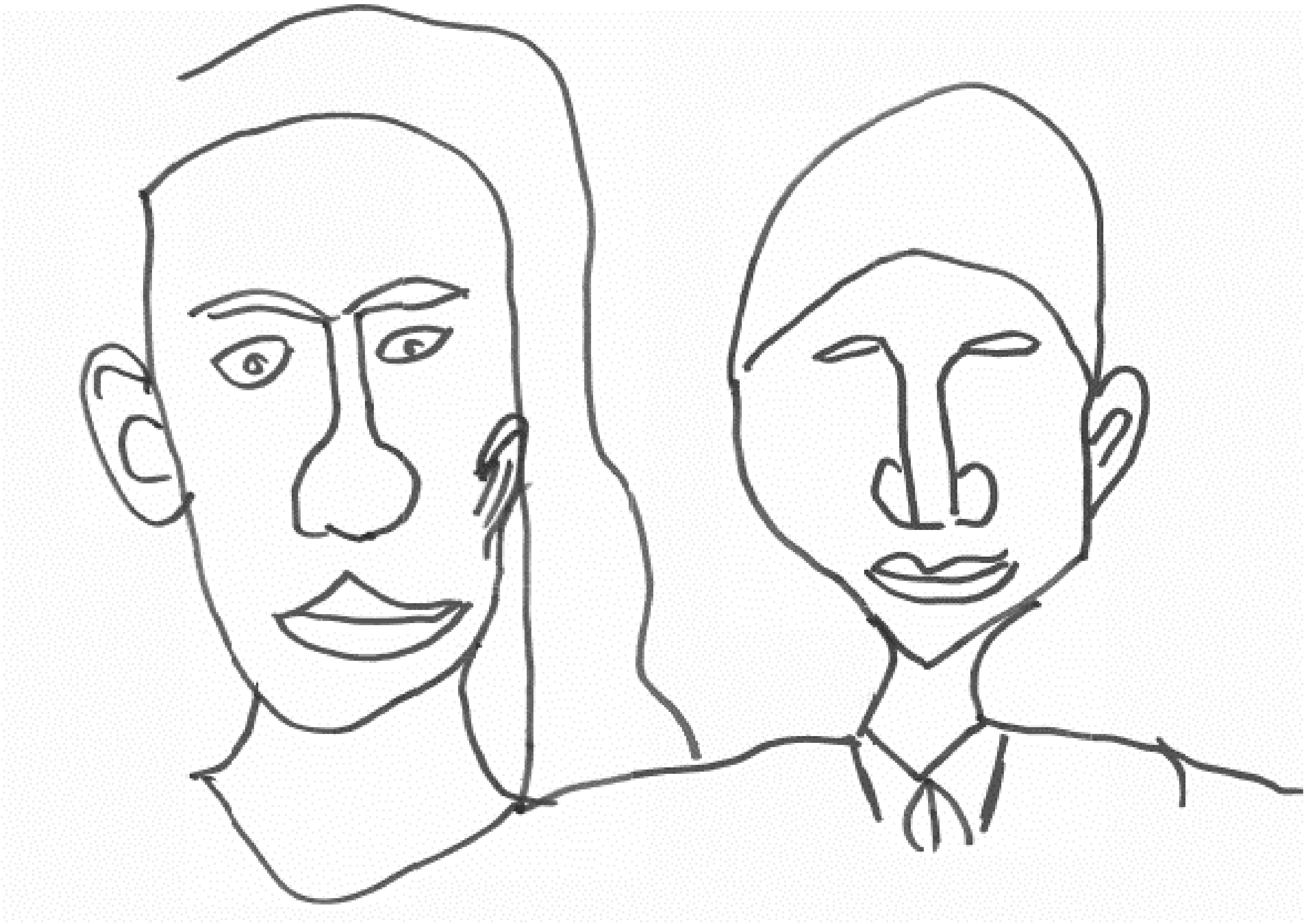

Case 3 Tracing Condition

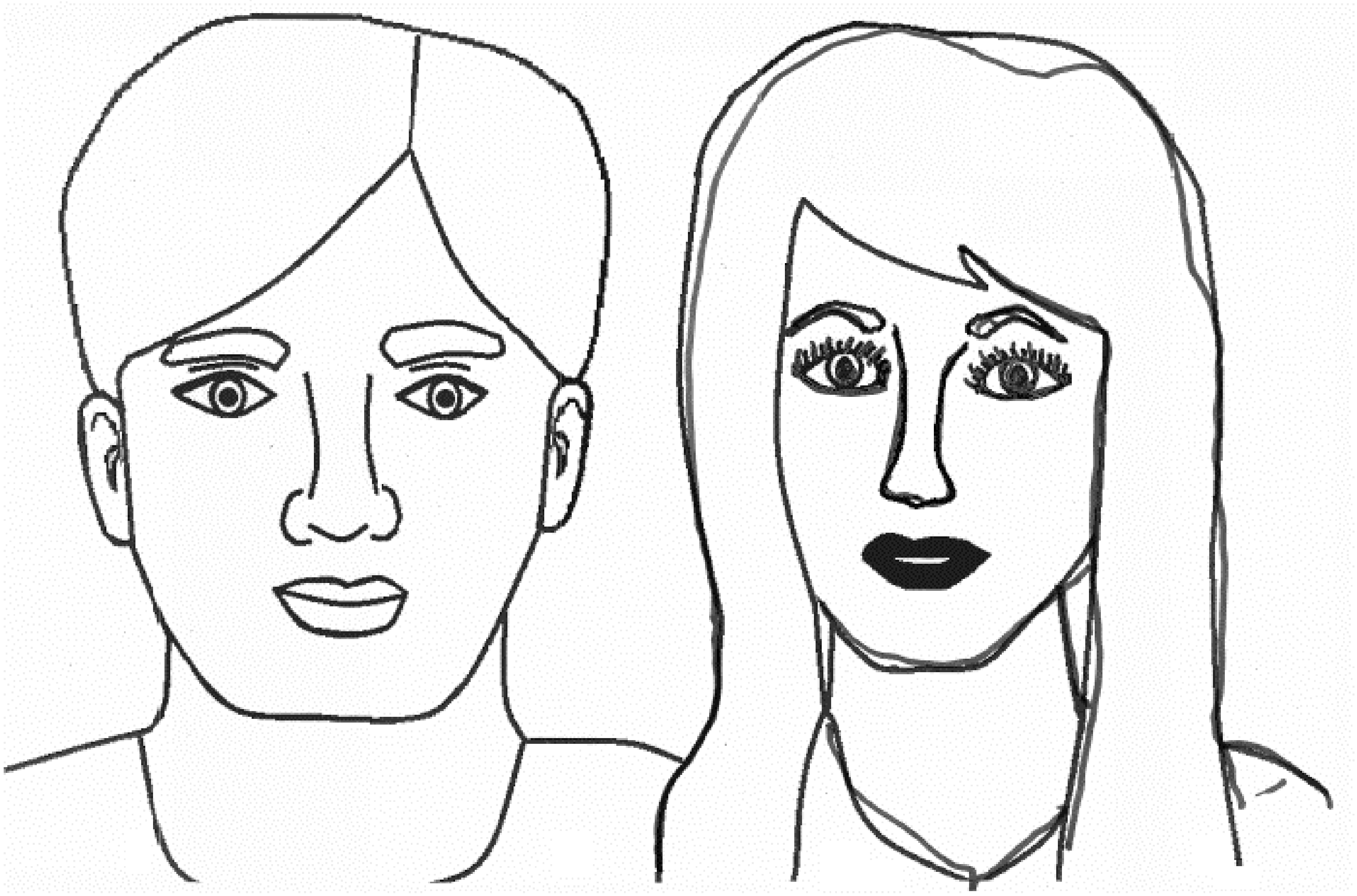

Case 4 Copying Condition

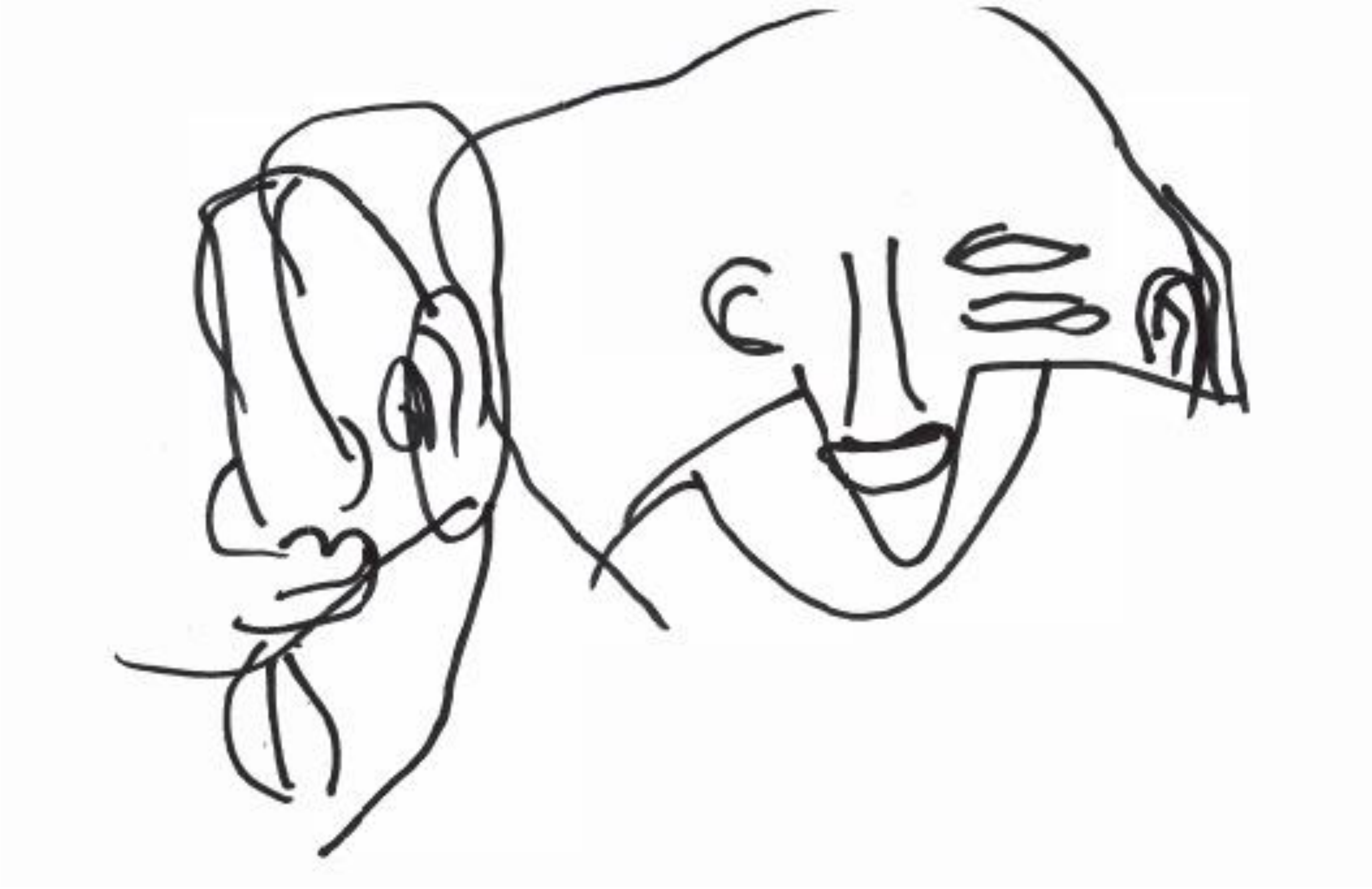

Case 4 Tracing Condition

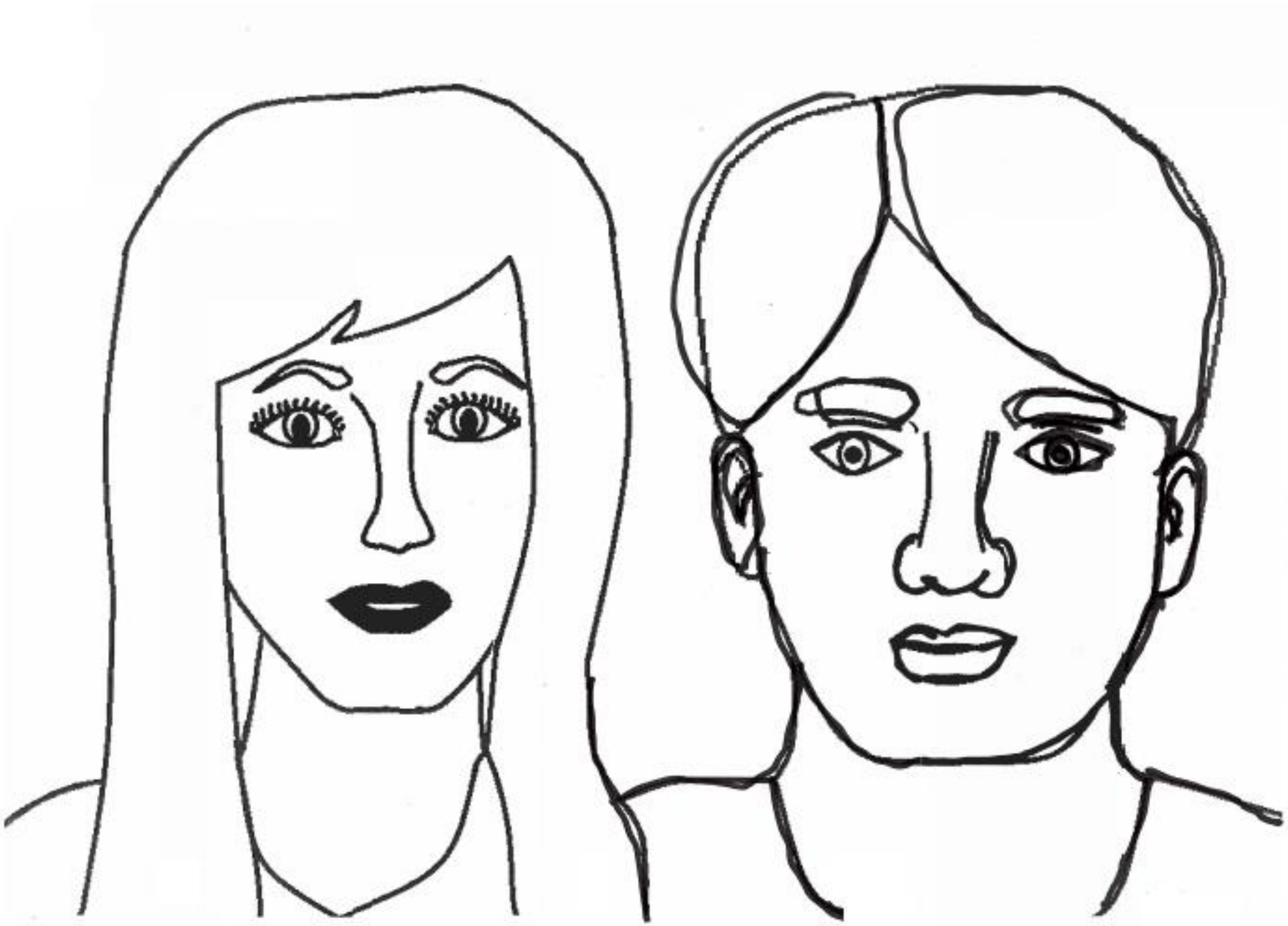

Case 5 Copying Condition

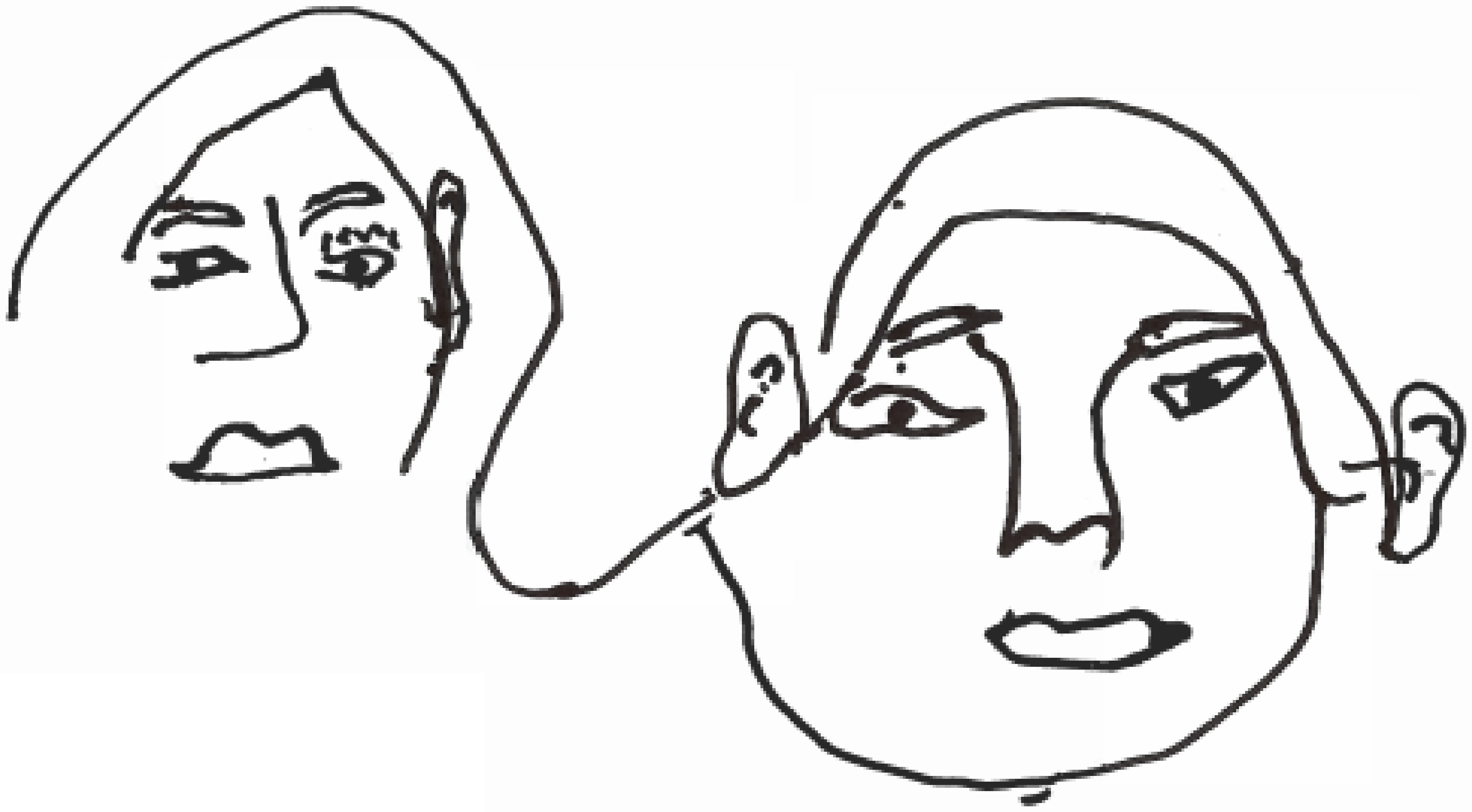

Case 5 Tracing Condition

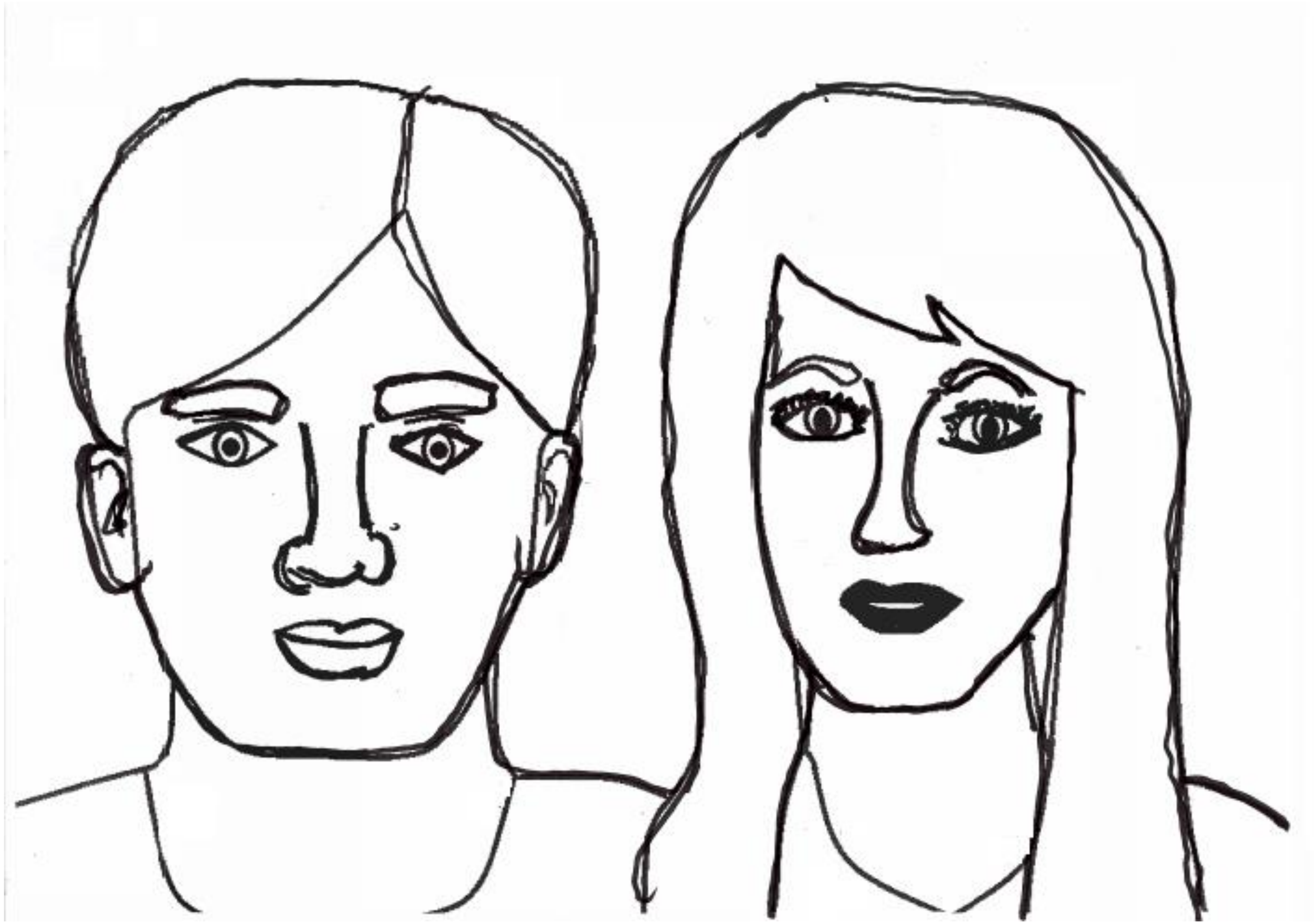

Case 6 Copying Condition

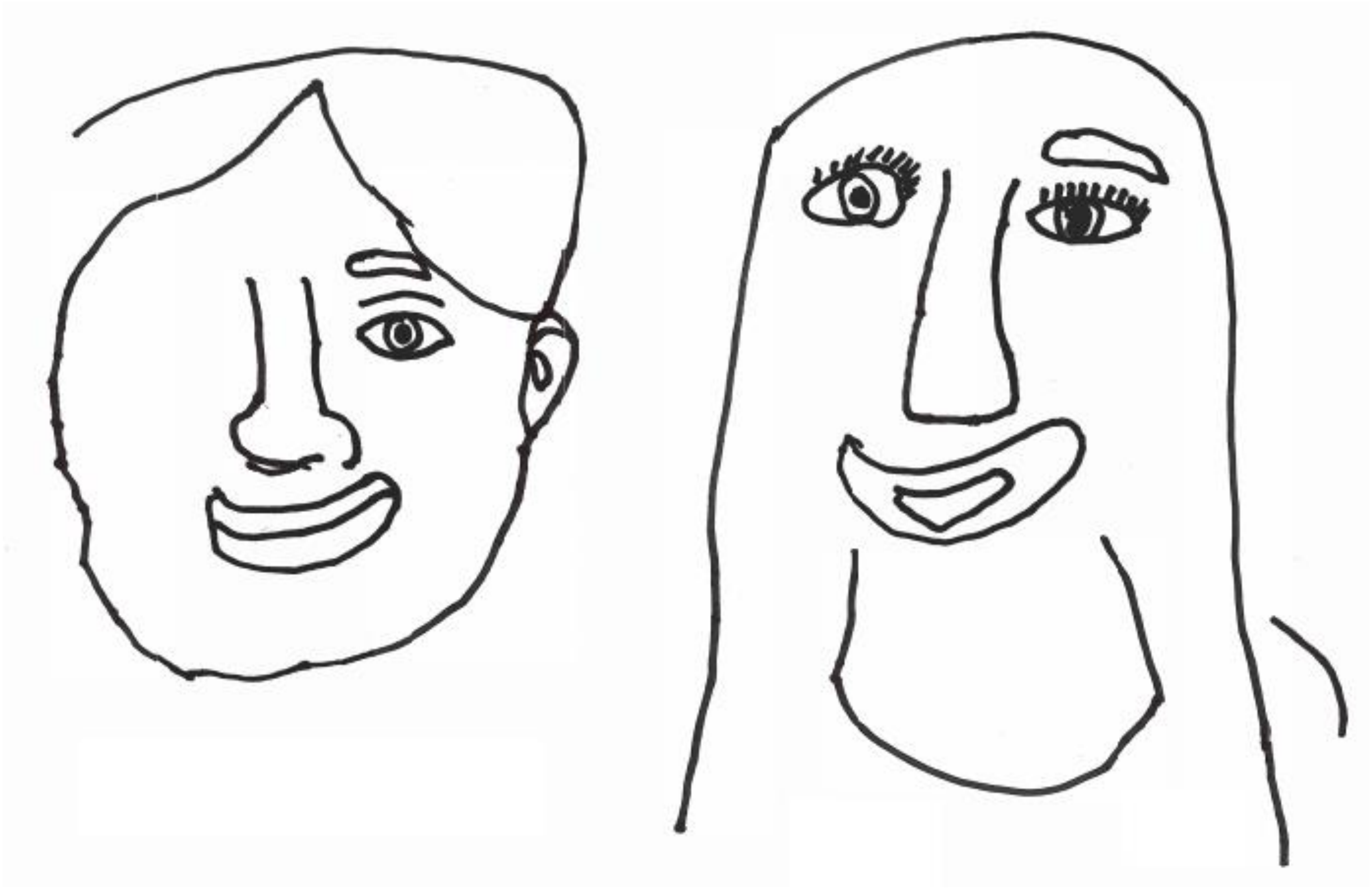

Case 6 Tracing Condition

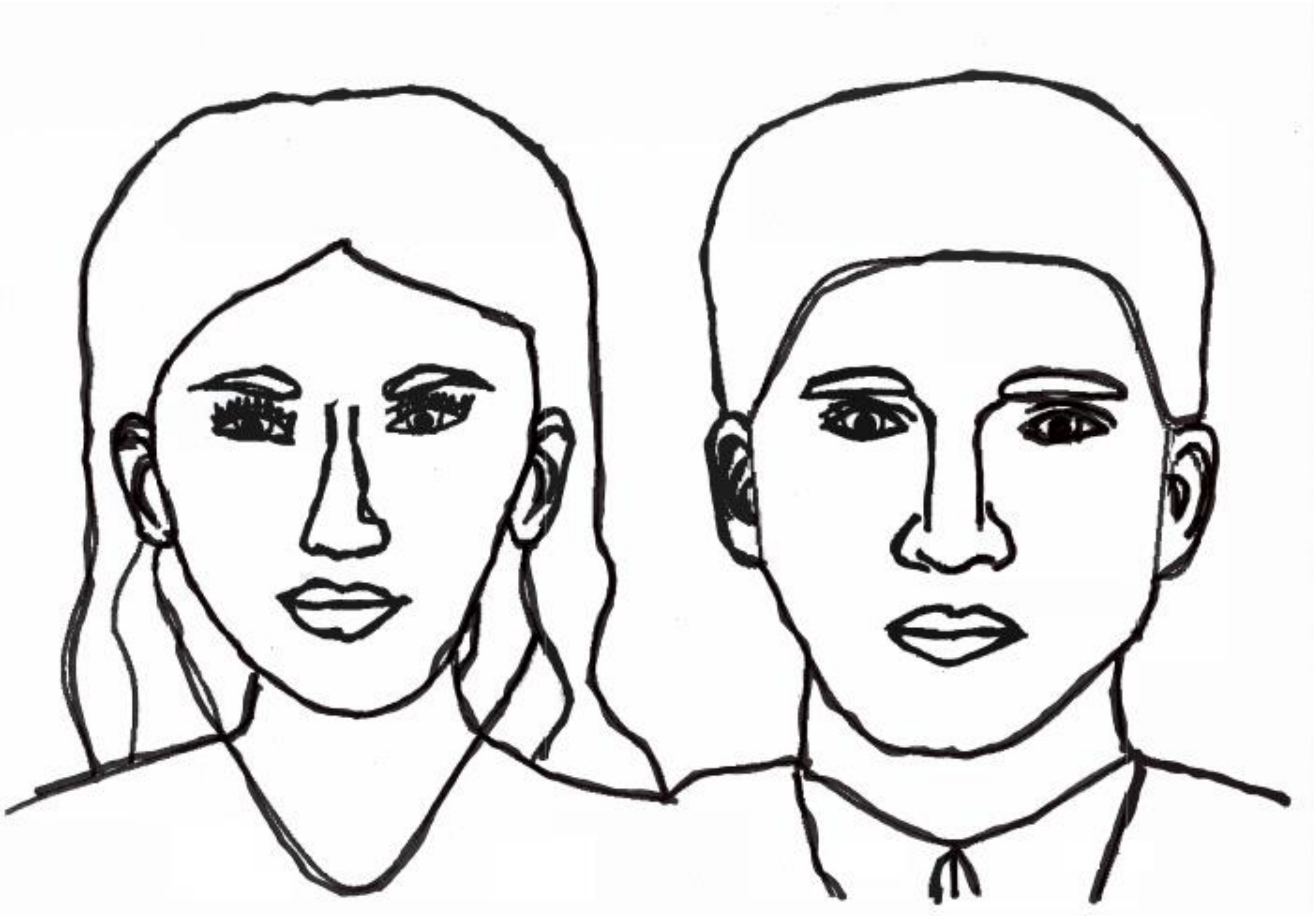

Supplement: Supplementary file 1 — Supplementary Information [file 41598_2017_2047_MOESM1_ESM.pdf]
